# Effect of Calcium Carbonate Fineness on Calcium Sulfoaluminate-Belite Cement

**DOI:** 10.3390/ma10080900

**Published:** 2017-08-03

**Authors:** Yeonung Jeong, Craig W. Hargis, Sungchul Chun, Juhyuk Moon

**Affiliations:** 1 Department of Civil and Environmental Engineering, National University of Singapore, 1 Engineering Drive 2, Singapore 117576, Singapore; ceejeon@nus.edu.sg; 2 Department of Construction Management, University of North Florida, 1 UNF Dr., Jacksonville, FL 32224, USA; craig.hargis@unf.edu; 3 Division of Architecture and Urban Design, Incheon National University, Incheon 22012, Korea

**Keywords:** green technology, sustainable concrete, carbon dioxide reduction, limestone, calcium sulfoaluminate, cement hydration

## Abstract

This study investigated the hydration characteristics and strength development of calcium sulfoaluminate-belite (CSAB) cements incorporating calcium carbonate (CC) powders with various particle size distributions and different gypsum amounts. In general, the CSAB hydration was accelerated by the CC powder, but the acceleration and resulting strength improvement were more effective with finer CC powder. Regardless of the fineness of the CC powder, it took part in the hydration of CSAB cement, forming hemicarboaluminate and monocarboaluminate phases. These hydration and nucleation effects compensated for the strength reduction from decreased cementing components (i.e., dilution effect) when finer CC powders were used, while they did not overcome the strength reduction when coarser CC powder was used. On the other hand, increasing the amount of gypsum for a given CC content improved the strength. The strength of CSAB cement had a clear inverse relationship with its total pore volume measured by mercury intrusion porosimetry (MIP). Thermodynamic modeling for CSAB cement hydration showed that the use of CC powder increased total volume of solid phases up to 6 wt % at a given amount of gypsum.

## 1. Introduction

Calcium sulfoaluminate (CSA) cements have received significant attention as an alternative cementitious binder to ordinary Portland cement (OPC) [1,2,3,4]. The use of CSA cement can be a possible solution to improve the sustainability of construction materials because of reducing fuel consumption and emitting less carbon dioxide (CO_2_) during manufacture. The production of CSA cement is achieved at relatively low temperatures near 1250 °C [2,3,4], while that of OPC requires higher temperatures near 1450 °C [5], implying that the manufacture of CSA cement is less energy-intensive than OPC. In addition, its production emits relatively less CO_2_ during calcination, compared to OPC production, due to the lower Ca content of CSA clinker [2].

A major constituent of CSA cement is ye’elimite [Ca_4_(AlO_2_)_6_·SO_4_ or C_4_A_3_$\bar{S}$ in cement chemistry notation] which comprises around 30–70 wt % of the CSA cement [3]. Note that in cement chemistry notation, C = CaO, S = SiO_2_, A = Al_2_O_3_, F = Fe_2_O_3_, T = TiO_2_, M = MgO, $\bar{S}$ = SO_3_, $\bar{C}$ = CO_2_, and H = H_2_O. Its hydration is generally fast compared to OPC hydration, and produces aluminum hydroxide [Al(OH)_3_], monosulfate (C_4_A$\bar{S}$H_12_), and ettringite (C_6_A${\bar{S}}_{3}$H_32_) depending on the added gypsum (C$\bar{S}$H_2_) content [4,6,7,8]. The molar ratio of calcium sulfate to ye’elimite (*m*-value) plays an important role in the type of its hydration products and dimensional stability of CSA based cements [2,4,6,7,8,9,10]. Winnefeld and Barlag [9] as well as Zhang [11] described that CSA cement could be categorized into three groups according to its *m*-value; (1) ‘rapid hardening/high strength’ with *m* < 1.5, (2) ‘expansive’ with 1.5 < *m* < 2.5, and (3) ‘self-stressing’ with *m* > 2.5. In addition to the *m*-value, the hydration and characteristics of CSA cements are also highly influenced by the water-to-cement ratio [10], thermal history [12], use of fillers [13,14] or supplementary cementitious materials (SCMs) [15,16], and blending with other cements [17,18].

Limestone, which mainly consists of calcium carbonate (CC) as the mineral calcite, is a main raw material for cement production [5,19] as well as an effective additive for cementitious pastes, mortars, and concrete [20,21,22,23,24,25,26]. It had been originally regarded as an inert filler due to the low solubility [20,21,27,28,29], but some researchers [25,26] reported positive chemical reactions induced from the limestone addition. The CC powder can modify the type of AFm phases (Al_2_O_3_-Fe_2_O_3_-mono) from monosulfate to hemicarboaluminate and/or monocarboaluminate phases with additional formation of ettringite, one of AFt (Al_2_O_3_-Fe_2_O_3_-tri) phases. This reaction increases the total volume of solid phases and improves the compressive strength of cementitious materials [7,25,26]. Many researchers have tried to use limestone in various cementitious systems such as OPC paste [25,30], SCM-blended OPCs [27,31,32], and alkali-activated cements [33]. In addition to the improvement of engineering properties, the use of limestone can also enhance the sustainability of construction materials by reducing the clinker component which consumes the most energy and emits the most CO_2_ during its manufacturing processes.

Several studies [13,14] were conducted to investigate the influence of limestone on the properties of CSA-based cements. Both studies reported that the use of limestone accelerated the hydration of CSA cements. Pelletier-Chaignat et al. [14] proposed that the use of limestone was more effective in improving the strength of CSA cement than that of quartz powder, which was consistant with results in OPC systems [28]. Martin et al. [13] found that at *m*-values below 2, limestone took part in the hydration reactions forming hemicarboaluminate and/or monocarboaluminate with additional ettringite, but the use of limestone was effective to improve the strength only in the case without a calcium sulfate source (i.e., *m* = 0). Although there has been a lot of research performed on the use of limestone in OPC systems, there has been relatively little research on the influence of limestone on CSA hydration and properties.

This study explores the hydration characteristics and materials properties of CSA-belite (CSAB) cements, blended with three CC powders with varying particle size distributions. Furthermore, the effect of changing the gypsum content for a fixed content of CC powder was studied by a series of experiments: X-ray diffraction (XRD), isothermal conduction calorimetry, compressive strength tests, mercury intrusion porosimetry (MIP), and hydration kinetics calculation.

## 2. Experimental Methods and Materials

### 2.1. Materials

Commercially available CSAB clinker and CC powder with different fineness were obtained from MIRAE C AND C Inc. (Yongin, Korea) and OMYA Malaysia Sdn. Bhd. (Shah Alam, Malaysia) respectively. All the materials were examined using XRF spectroscopy (S4 Pioneer, Bruker AXS GmbH, Karlsruhe, Germany), a laser diffraction particle size analyzer (Mastersizer 3000, Malvern Instruments Ltd., Malvern, UK) and XRD (LabX XRD-6000, Shimadzu Co., Kyoto, Japan). Reagent grade gypsum (98% purity, Samchun Chemical Co., Pyeongtaek, Korea) was also used for this study.

**Figure 1:**
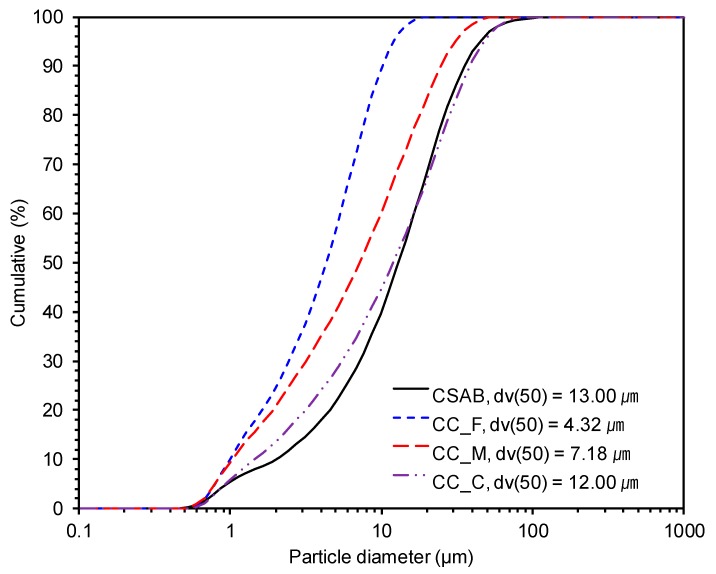
Particle size distribution of calcium sulfoaluminate (CSA)-belite clinker and three calcium carbonate powders with different fineness; CC indicates calcium carbonate powder, and F, M, and C indicate the finest, middle, and the coarsest powder, respectively; dv(50) shows the median value of each powder.

Particle size distributions of the raw materials are shown in Figure 1. The finest and coarsest CC powders were named as CC_F and CC_C, respectively, and the middle size of CC powder was labelled as CC_M. The CSAB clinker had a similar particle size distribution as CC_C, but was clearly coarser than CC_F and CC_M. Oxide chemical compositions and loss on ignition (LOI) of the CSAB clinker and CC powders are presented in Table 1. All CC powders had a large number of LOI values due to the loss of carbon dioxide in the CC powders.

**Figure 2:**
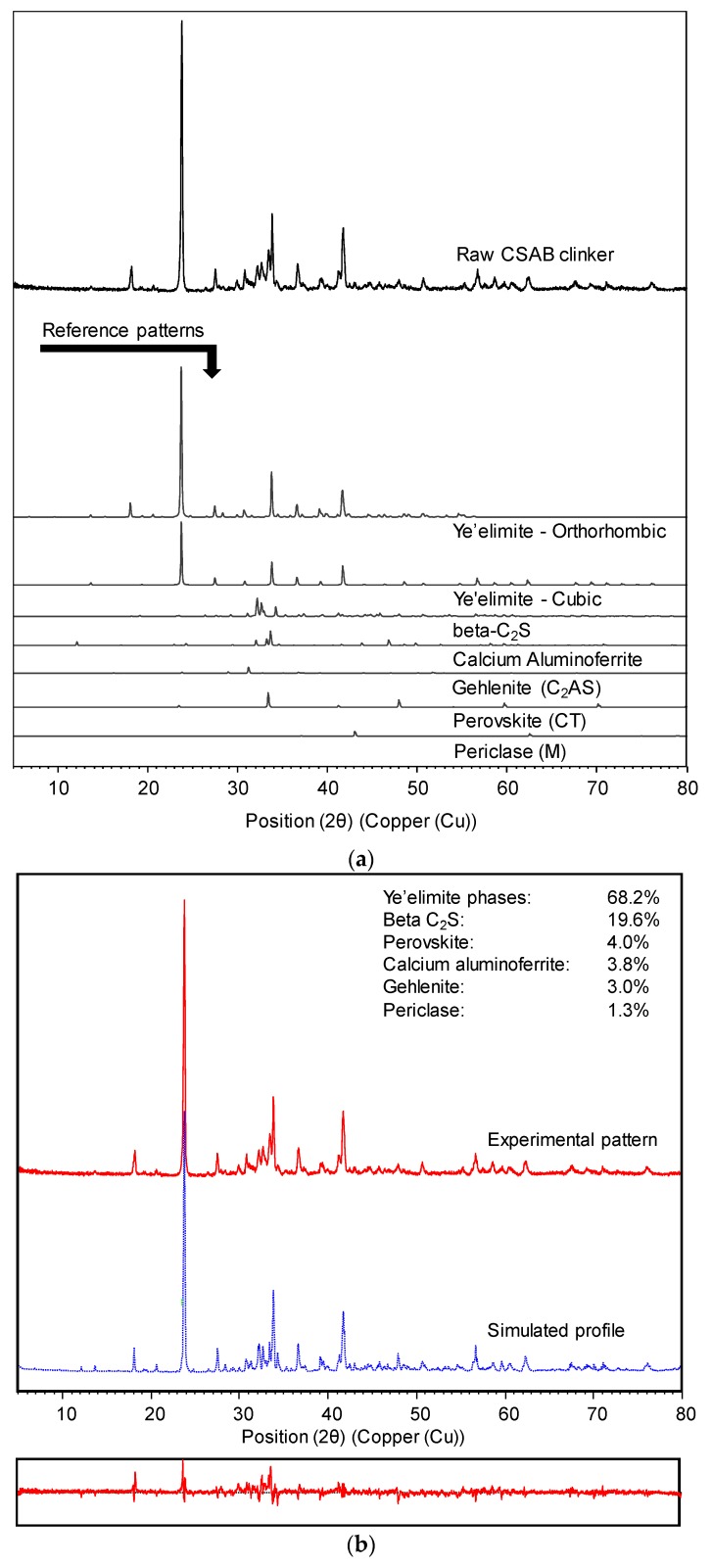
(**a**) Qualitative phase analysis and (**b**) quantitative X-ray diffraction (XRD) analysis with Rietveld refinement for the CSAB clinker.

Qualitative phase analysis (QPA) and Quantitative XRD (QXRD) analysis with Rietveld refinement were carried out for all raw materials. Details of XRD procedures will be explained in the next section. Figure 2a,b shows the QPA and QXRD results for the CSAB clinker used in this study. Two types of ye’elimite phases (cubic and orthorhombic) and beta-C_2_S (β-Ca_2_SiO_4_) were contained in the raw CSAB clinker as major components, and calcium aluminoferrite [Ca_2_(Al,Fe)_2_O_5_, C_4_AF], gehlenite [Ca_2_Al(AlSiO_7_), C_2_AS], perovskite (CaTiO_3_, CT), and periclase (MgO, M) were included as minor components. All the mineral phases were commonly known crystalline phases in CSAB cements [4,13,14,34,35]. No calcium sulfate sources such as anhydrite (CaSO_4_) or gypsum (CaSO_4_·2H_2_O) were identified in the raw CSAB clinker. Table 2 presents all the results of QXRD for CSAB clinker and CC powders including the results of Figure 2b. All CC powders consisted of principally calcite (CaCO_3_, C$\bar{C}$) and dolomite [CaMg(CO_3_)_2_, CM$\bar{C}$_2_] as an impurity. The purity of the CC powder increased with fineness.

### 2.2. Experimental Details

Raw CSAB clinker was blended with 10.5% gypsum to produce a CSAB cement with an *m* value of 0.65. The amount of CSAB cement was substituted by 5 wt % of CC powder with varying fineness to investigate the influence of CC fineness on the properties of CSAB cement pastes with a constant *m* value. For the sample with CC_M, the substitution amount of gypsum was raised from 10 wt % to 15 wt % and 20 wt %, respectively, to examine the effect of varying gypsum content with a given amount of CC powder. The water-to-powder ratio was 0.5 for all samples. Table 3 summarizes the details of the mixture proportions.

All CSAB cements were dry-mixed by hand for 5 min, and then were placed into a mixing bowl. De-ionized water was poured into the CSAB cements and mixed by a Hobart mixer on slow speed, around 140 rpm, for 30 s and remixed on medium speed, around 285 rpm, for 60 s. The mixed pastes were cast into 50 × 50 × 50 mm cubic molds for compressive strength tests, and ϕ25.4 × 25.4 mm cylinder molds for MIP and XRD experiments. All pastes were cured at a constant temperature of 30 ± 2 °C and 65% relative humidity for 24 h prior to de-molding. The samples at 24 h were sealed by a plastic vinyl sheet and cured again under the same curing conditions until each testing-day.

The hydration heat release of each sample was measured using an isothermal conduction calorimeter (TAM Air, TA Instruments Co., New Castle, DE, USA) at 30 °C for 90 h. Before 1 day of calorimetric analysis, blended cements and mixing water were stored in an oven at 30 °C to shorten equilibrium time. Freshly mixed pastes prepared from Table 3 were placed into glass vials as approximately 10 ± 1 g and its isothermal calorie was measured, simultaneously, in each channel of the instrument. Measured results were normalized by sample weight.

Compressive strength tests were carried out at curing ages of 1, 3, 7, 14, and 28 days. For each sample, three samples were tested and independent results were averaged to determine the strength of the samples. The samples cast in ϕ25.4 × 25.4 mm cylinder molds were sliced into cube pieces of 5 × 5 × 5 mm for MIP measurements, and were crushed into powder for XRD measurements. The hydration of MIP and XRD samples was stopped using isopropyl alcohol (IPA), followed by rinsing with diethyl ether and a short drying period at 40 °C to remove the remaining diethyl ether [31,36,37].

The XRD patterns of raw materials and hardened pastes were collected using an X-ray diffractometer (LabX XRD-6000, Shimadzu Co., Kyoto, Japan) with Cu-Kα radiation (λ = 1.5418 Å) and a scanning range of 5° to 80° 2θ. Measured patterns were analyzed using the PANalytical X’pert HighScore Plus software (version 3.0.5) [38] with the Inorganic Crystal Structure Database (ICSD) [39] and the Crystallography Open Database (COD) [40]. For raw materials, QXRD using Rietveld refinement was performed by refining the phase scale factors, unit cell parameters, peak profile asymmetry, zero shift, and specimen displacement. Backgrounds of the patterns were manually determined and fixed during the refinement process. No amorphous phases were considered for the raw materials. As shown in Figure 2b, the simulated pattern matched well and obtained an R_exp_ value below 8.7.

The pore size distribution of each hardened paste at 28 days was investigated with a mercury porosimeter (AutoPore IV 9500, Micromeritics Instrument Co., Norcross, GA, USA) under a pressure range from 0.10 to 60,000.00 psia.

Thermodynamic modeling was performed using the Gibbs Energy Minimization Software (GEMS, version 3) developed and maintained by the Paul Scherrer Institute [41,42]. The thermodynamic data for aqueous species and solids were taken from the GEMS thermodynamic database with the PSI-Nagra database [43]. For cement minerals the cement-specific CEMDATA14 database [44,45,46] was used.

To examine the chemical effects of adding CC powder to the CSAB cement, thermodynamic modeling was utilized to determine the long term effect of changing the amount of calcium carbonate in the CSAB cement on the hydration phase assemblage and the total solid volume. The CSAB clinker and fine calcium carbonate powder compositions used in the modeling were taken from the Rietveld refinement. The CSAB clinker was combined with 10 wt % gypsum, then the amount of fine calcium carbonate powder was varied from 0 to 15 wt %. The model allowed for an Al-Fe solid solution in monosulfate and a SO_4_-CO_3_^2−^ solid solution in ettringite. Additionally, both Al and Fe monocarbonate were utilized [47]. The minor phases of perovskite, gehlenite, and periclase were considered inert due to their observed low reactivity in other CSA systems and a lack of strong evidence to the contrary in the present study. Ye’elimite, larnite, and brownmillerite were allowed to hydrate fully.

## 3. Results and Discussion

### 3.1. Isothermal Conduction Calorimetry

**Figure 3:**
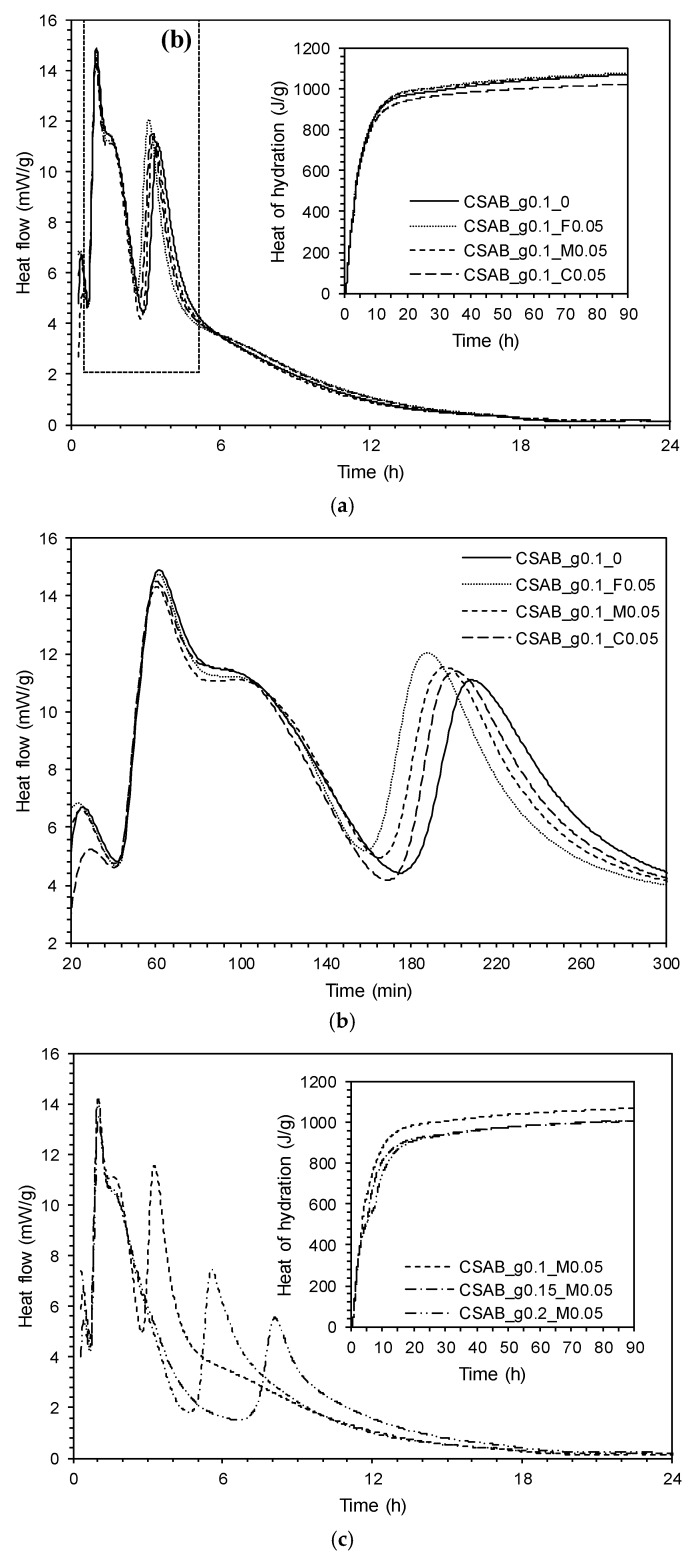
Isothermal conduction calorimetry of each sample: (**a**) influence of calcium carbonate powder with different fineness; (**b**) magnification of dashed-box area in (**a**); and (**c**) influence of gypsum content under the presence of calcium carbonate powder.

The isothermal conduction calorimetry results are shown in Figure 3a–c. The first main hydration peaks occurred at about 1 h with a shoulder after approximately 2 h in all samples. The time for the first peak and shoulder was not influenced by the presence of CC and gypsum. The second major peak of CSAB_g0.1_0 occurred after 220 min, but occurs earlier in pastes containing CC powder as shown in Figure 3b. In addition, the acceleration of the second peak of hydration increased with increasing fineness of the CC powder, indicating a nucleation effect of the CC powder on the CSAB cement. Previous studies have mentioned that CC powder could accelerate the hydration of alkali-activated slag-fly ash blends [33], pure ye’elimite [7], CSA cement blended with gypsum [13], Portland cement [25], and fly ash-blended Portland cement [22] by providing additional nucleation sites for hydration products as a filler. In this study, the finer CC powder was more effective in accelerating the hydration of CSAB cement than coarser CC powder, likely due to supplying more nucleation sites. An increase in calcium sulfate content slightly decreased the intensity of the first peak. However, it significantly delayed the appearance of the second peak and lowered its intensity with increasing calcium sulfate content as shown in Figure 3c. Jansen et al. [48] studied the hydration of cubic and orthorhombic ye’elimite blended with gypsum and reported similar hydration heat profiles with this study. Chen and Juenger [49] attributed the second peak to the dissolution and/or reaction of ye’elimite after initial gypsum depletion, indicating that more gypsum content could delay the occurrence of the second peak. The results of current work are consistent with the previous studies.

The total heat of hydration after 90 h was 1070 J/g for CSAB_g0.1_0, 1077 J/g for CSAB_g0.1_F0.05, 1068 J/g for CSAB_g0.1_M0.05, 1023 J/g for CSAB_g0.1_C0.05, 1006 J/g for CSAB_g0.15_M0.05, and 1010 J/g for CSAB_g0.2_M0.05. The sample CSAB_g0.1_M0.05 containing CC_M had similar total hydration heat compared with the control sample (i.e., CSAB_g0.1_0). The sample containing finer CC powder (i.e., CC_F) had more hydration heat, while the sample containing coarser CC powder (i.e., CC_C) released less hydration heat than CSAB_g0.1_0. The results indicate that CC powder having similar fineness with CSAB cement (in this study, CC_C) decreases the total hydration heat, but the CC powders with the finer particle sizes than the CSAB cement (CC_M and CC_F) generate more hydration heat. Previous studies [27,50] reported that the hydration heat decreased when some fractions of cementing components were substituted by less reactive supplementary materials such as GGBFS, fly ash, and limestone, which was induced from the decreased amount of clinker components and was referred to as a dilution effect [51]. Although only 5 wt % of CSAB cement was replaced by CC powder, total hydration heat after 90 h was equivalent or higher than that of the control sample when finer CC powders than the CSAB clinker were incorporated (i.e., CSAB_g0.1_M0.05 and CSAB_g0.1_F0.05). This result confirms that the finer limestone filler yields more hydration reaction even with its dilution effect. The finer CC powder supplies more surface areas as nucleation sites for hydration products [28,29], yielding the larger hydration heat release at early ages.

### 3.2. Compressive Strength Tests

**Figure 4:**
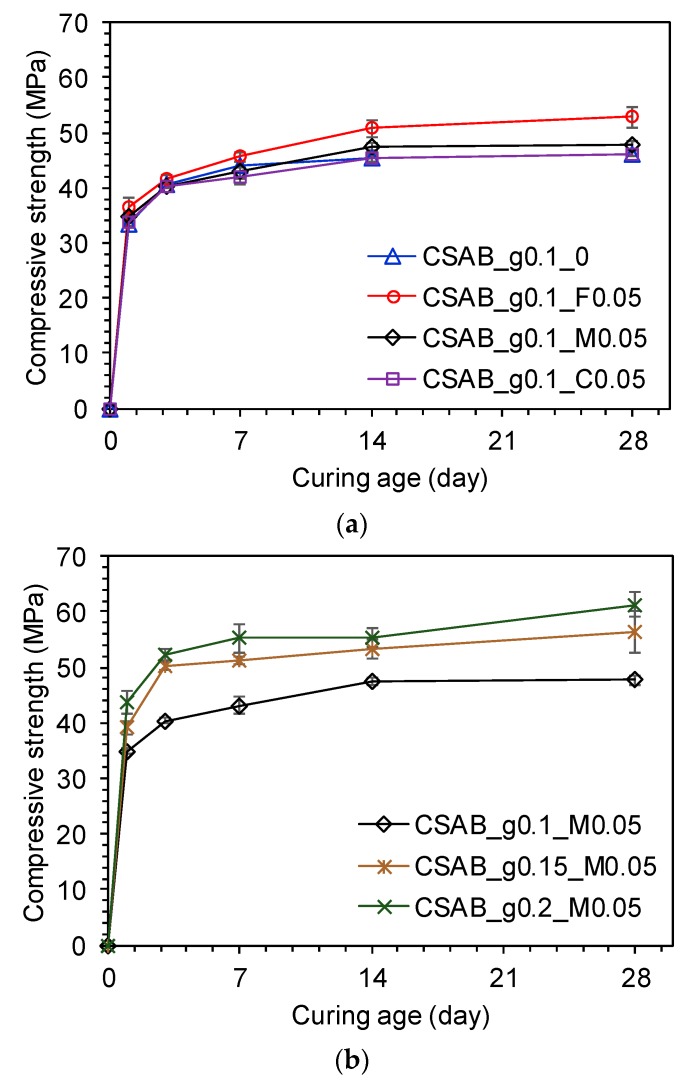
Strength development of each paste depending on (**a**) different CC fineness and (**b**) gypsum content in the presence of CC powder.

Results of compressive strength tests are presented in Figure 4. The results show that the use of CC_F and CC_M improve the strength of the pastes, the enhancement becomes more significant with increasing ages, and the pastes with the finest limestone have higher strengths at all ages, which is consistent with nucleation benefits at early ages and more calcium carbonate participating in hydration reactions at later ages [7,14]. However, the use of CC powder with similar fineness as the CSAB clinker did not improve its strength. As explained previously, the higher fineness provided more surface area for hydration product nucleation. This led to an acceleration of hydration and an improvement of mechanical properties [28,29,51]. However in the case of the CSAB_g0.1_C0.05 sample, the compressive strength was almost identical to that of the control sample, CASB_g0.1_0. This indicates that the positive effect of using CC filler on compressive strength might not have overcome the dilution effect when the CC powder of similar fineness to the CSAB clinker was used.

As shown in Figure 4b, increasing the gypsum content improved the strength of the CSAB paste with a given amount of CC powder (i.e., 5 wt % of CC_M). Previous studies have proposed that gypsum plays an important role in controlling the hydration of ye’elimite, a main component of CSAB cements, and the dimensional stability of CSA based cements [4,7,9] based on the following chemical reactions (1) and (2).

(1) $$C_{4}A_{3}\bar{S}+18H\to C_{3}A\cdot C\bar{S}H_{12}+2{\mathrm{AH}}_{3}\;\left(\mathrm{monosulfate}\;\mathrm{formation}\;\mathrm{without}\;\mathrm{gypsum}\right)$$

(2) $$C_{4}A_{3}\bar{S}+2C\bar{S}H_{2}+34H\to C_{3}A\cdot 3C\bar{S}H_{32}+2{\mathrm{AH}}_{3}\;\left(\mathrm{ettringite}\;\mathrm{formation}\;\mathrm{with}\;\mathrm{gypsum}\right)$$

In addition, García-Maté et al. [52] stated that ettringite formation was a key factor for strength development of commercial CSA cement. In this study of CSAB with CC powder, it could be similarly expected that the increase in gypsum content led to the strength enhancement due to more formation of ettringite.

### 3.3. Powder X-ray Diffraction Analysis

**Figure 5:**
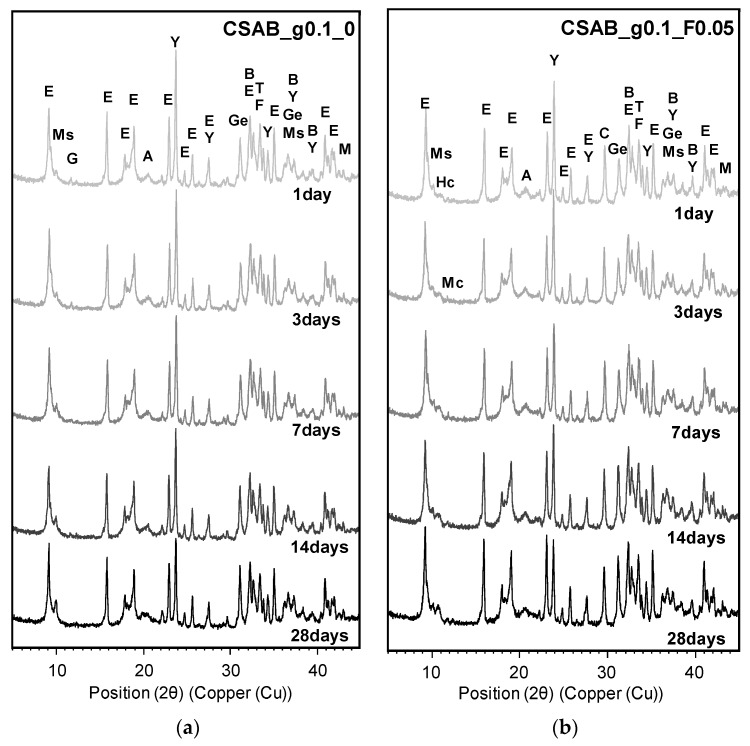

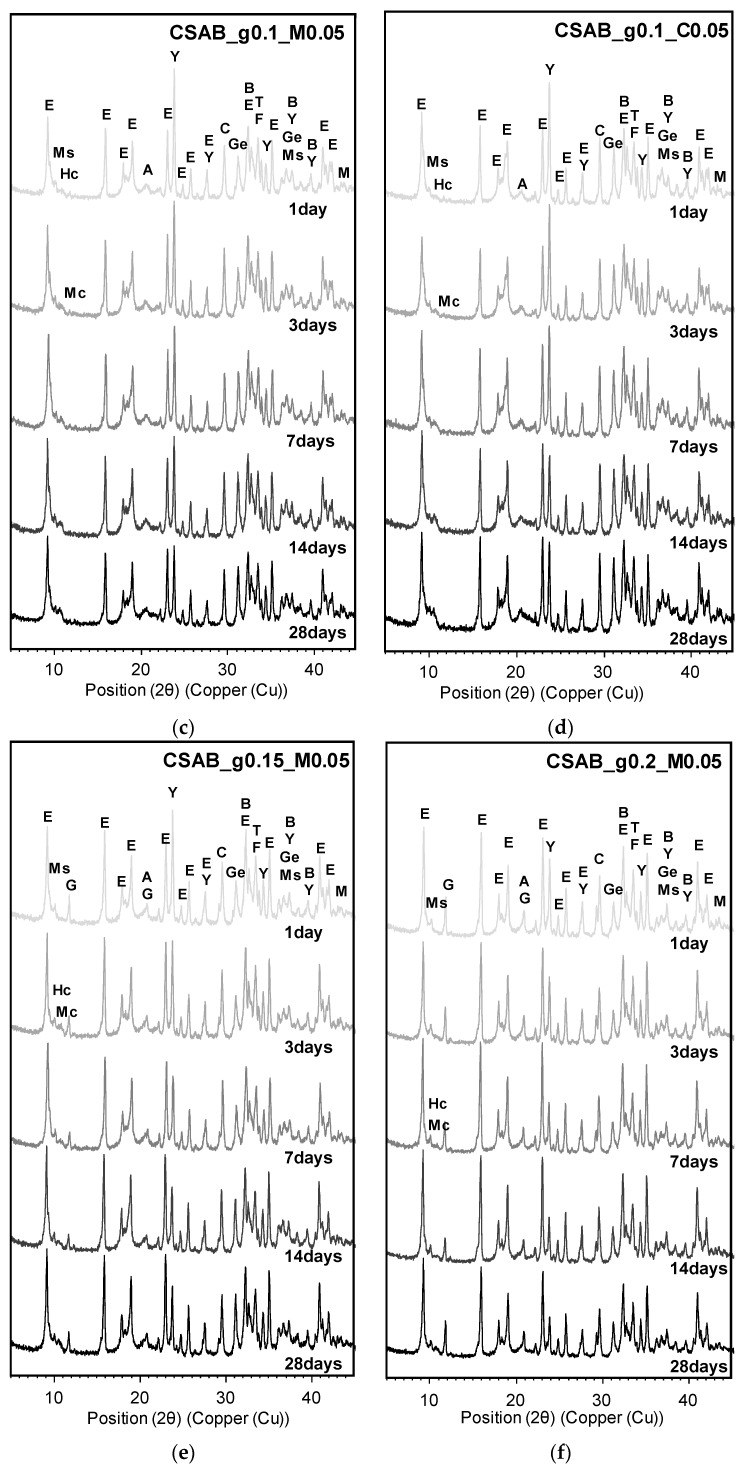
Measured X-ray diffraction patterns: E indicates ettringite, Ms monosulfate, Hc hemicarboaluminate, Mc monocarboaluminate, G gypsum, A amorphous aluminum hydroxide [Al(OH)_3_], Y ye’elimite, C calcite, Ge gehlenite, B beta-C_2_S, T CaTiO_3_, F calcium aluminoferrite, and M MgO. (**a**) CSAB_g0.1_0; (**b**) CSAB_g0.1_F0.05; (**c**) CSAB_g0.1_M0.05; (**d**) CSAB_g0.1_C0.05; (**e**) CSAB_g0.15_M0.05; (**f**) CSAB_g0.2_M0.05.

Measured XRD patterns of each sample at 1, 3, 7, 14, and 28 days are presented in Figure 5a–f. Residual crystalline phases originally contained in the CSAB clinker were identified in all samples. This was because of the relatively short hydration period (i.e., 28 days) and low water-to-cement ratio (i.e., 0.5) chosen in this study. In addition, calcite reflections were also identified in all samples with CC powder regardless of its fineness and gypsum content. This was due to the low solubility and slow dissolution rate of the CC powder [25,26,30,32]. 

The sample of CSAB_g0.1_0 which did not contain CC powder produced amorphous aluminum hydroxide [Al(OH)_3_], monosulfate, and ettringite [Ca_6_Al_2_(SO_4_)_3_(OH)_12_·32H_2_O, C_6_A${\bar{S}}_{3}$H_32_] as hydration products, which were common hydration products of CSA and CSAB cements [1,2,4,9,13]. In addition to those products, hemicarboaluminate [Ca_4_Al_2_(OH)_12_·OH(CO_3_)_0.5_·4H_2_O, C_4_A${\bar{C}}_{0.5}$H_10.5_] and monocarboaluminate [Ca_4_Al_2_(OH)_12_(CO_3_)·5H_2_O, C_4_A$\bar{C}$H_11_] phases formed in the samples containing CC powders and those reflections became clearer with time, implying that CC powders gradually reacted regardless of the CC powder fineness and gypsum amount. Those phases have been frequently identified in various cementitious materials containing CC [13,25,30,31,32] because the addition of CC can generate hemicarboaluminate and/or monocarboaluminate with additional formation of ettringite [25,26]. Equations (3) and (4) give net chemical reactions for the formation of monocarboaluminate and hemicarboaluminate directly from cement phases, and Equations (5) and (6) present net chemical reactions for the conversion of monosulfate to monocarboaluminate and hemicarboaluminate with the production of additional ettringite. Whereas the formation of monocarboaluminate is thermodynamically preferred to that of hemicarboaluminate, hemicarboaluminate has been frequently formed before the formation of monocarboaluminate because of the slow dissolution of CC powder and relatively fast formation of hemicarboaluminate [25,31].

(3) $$3C_{4}A_{3}\bar{S}+2C\bar{C}+72H\to 2C_{4}{A\bar{C}H}_{11}+C_{6}{A\bar{S}}_{3}H_{32}+6{\mathrm{AH}}_{3}$$

(4) $$6C_{4}A_{3}\bar{S}+C\bar{C}+132H\to 2C_{4}{A\bar{C}}_{0.5}H_{10.5}+2C_{6}{A\bar{S}}_{3}H_{32}+14{\mathrm{AH}}_{3}+5\mathrm{CH}$$

(5) $$3C_{4}{A\bar{S}H}_{12}+2C\bar{C}+18H\to 2C_{4}{A\bar{C}H}_{11}+C_{6}{A\bar{S}}_{3}H_{32}$$

(6) $$3C_{4}{A\bar{S}H}_{12}+C\bar{C}+\mathrm{CH}+16H\to 2C_{4}{A\bar{C}}_{0.5}H_{10.5}+C_{6}{A\bar{S}}_{3}H_{32}$$

The phase transformation from monosulfate to carbonated AFm phases with additional ettringite can improve the mechanical strength of various cementitious materials by increasing total volume of solids [13,25,26,32,53]. In addition, the formation of the carbonated AFm phases can improve the stability of ettringite formed, at later ages, by supplying additional SO_4_^2−^ anions from monosulfate [30]. In this study, the strength improvement with CC powder was found in the CSAB_g0.1_F0.05 and CSAB_g0.1_M0.05 samples which contain CC powders finer than the CSAB clinker (i.e., CC_F and CC_M), while there was no clear strength improvement in the CSAB_g0.1_C0.05 sample in which coarser CC powder was used (i.e., CC_C). The reason why the use of CC_C powder did not improve the strength of CSAB pastes could be due to the previously explained dilution effect. Even though the total solid volume was increased as a result of the formation of carbonated AFm phases as well as additional ettringite, the amount of CSAB cement was decreased by the CC powder substitution. This could be a negative effect on strength development. Nevertheless, the use of CC_F and CC_M enhanced the strength of CSAB pastes. This is clearly due to the acceleration and nucleation effects that may compensate for the dilution effect. (See Figure 3a where the total hydration heats of CSAB_g0.1_F0.05 and CSAB_g0.1_M0.05 were slightly higher and almost identical, while that of CSAB_g0.1_C0.05 was clearly lower, compared to that of CSAB_g0.1_0) With the coarsest CC powder, the dilution effects balanced the nucleation and participatory chemical reactions benefits of the CC powder, resulting in strengths that were similar to the CSAB paste without CC.

Residual gypsum reflections were identified in CSAB_g0.1_0, CSAB_g0.15_M0.05, and CSAB_g0.2_M0.05 samples as shown in Figure 5a,e,f. In CSAB_g0.15_M0.05 and CSAB_g0.2_M0.05, much gypsum was added into pastes, so some residuals did not participate in the hydration of CSAB cement due to the short hydration period (i.e., 28 days) and relatively low water-to-cement ratio (i.e., 0.5). Whereas the other CSAB_g0.1 series with CC powder did not show the gypsum reflections, the measured XRD pattern of CSAB_g0.1_0 indicated small, but clear gypsum reflections. The hydration of the CSAB_g0.1 group with CC powder was accelerated by CC powder as shown in Figure 3b, causing complete depletion of added gypsum regardless of the fineness of CC powders.

### 3.4. Mercury Intrusion Porosimetry

**Figure 6:**
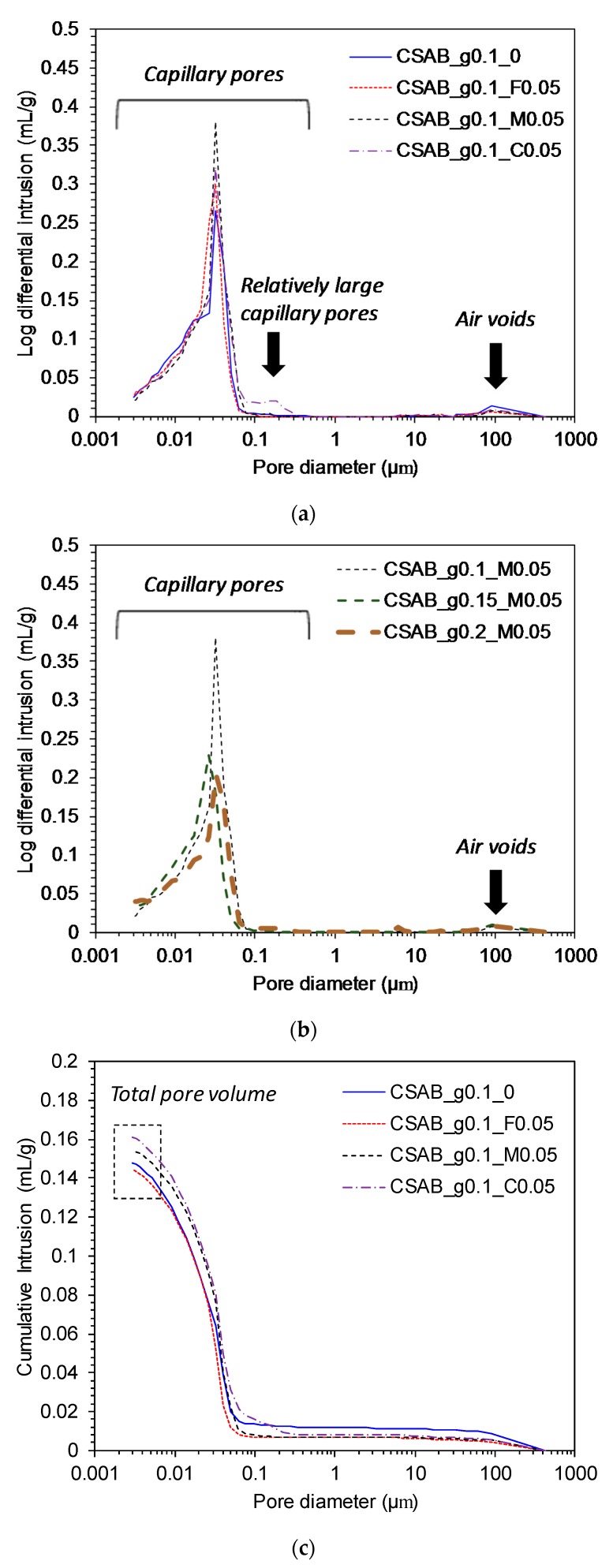

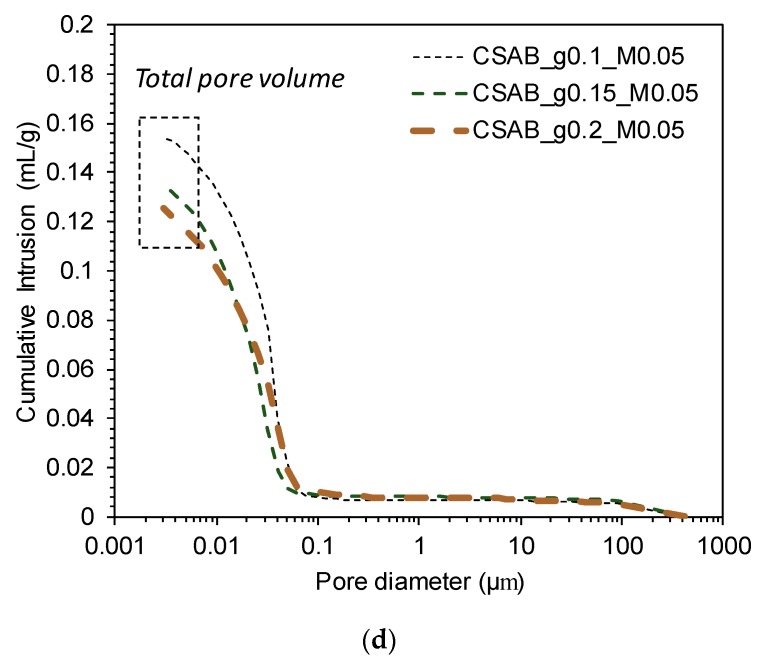
Results of mercury intrusion porosimetry at 28 days; (**a**,**b**) present pore size distributions; (**c**,**d**) show cumulative pore volumes.

Pore distributions and cumulative pore volumes of each paste at 28 days are presented in Figure 6a–d. As shown in Figure 6a,b, the pores of all pastes can be roughly divided into two parts which are capillary pores below 1 μm and entrained air voids larger than 60 μm [19]. In general, larger pores influences the strength of cementitious materials and smaller pores relate to creep and shrinkage behaviors [19,54,55].

Only the CSAB_g0.1_C0.05 sample contained relatively large sized capillary pores around 0.2 μm while the other samples predominately included pores smaller than 0.08 μm. In addition, CSAB_g0.1_C0.05 had a larger total pore volume than the others. These things can help to explain the lower strength development of CSAB_g0.1_C0.05 compared to the other samples. Increasing the gypsum content significantly reduced capillary pore volumes as shown in Figure 6b, which was consistent with the results of compressive strength in Figure 4b because pore volumes generally had an inverse relationship with compressive strength [19].

**Figure 7:**
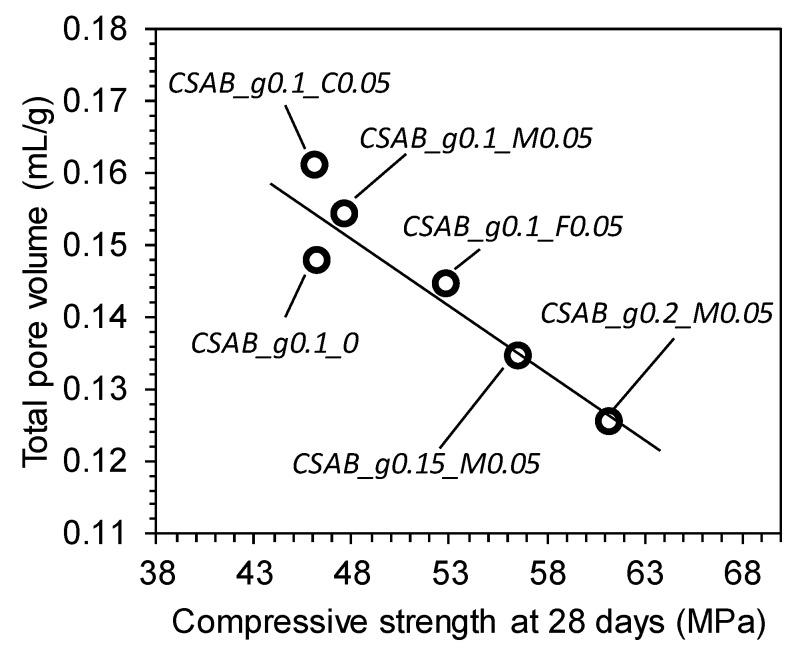
Relationship between compressive strength and total pore volumes of each paste at 28 days.

The relationship between compressive strength and total pore volume at 28 days is presented in Figure 7. Although previous studies [54,56,57] insisted that total pore volume from MIP may not reflect real pore structures of cementitious materials due to the sample damage by high pressure, the ink-bottle effect, and residual unreacted particles, Figure 7 presents a clear inverse relationship between the total pore volume and compressive strength. As a result of this MIP study, it can be confirmed that the total pore volume could be a proper indicator for compressive strength of CSAB cements, similarly reported by other studies [10,19,32,53,58].

### 3.5. Modeling of Cement Hydration

**Figure 8:**
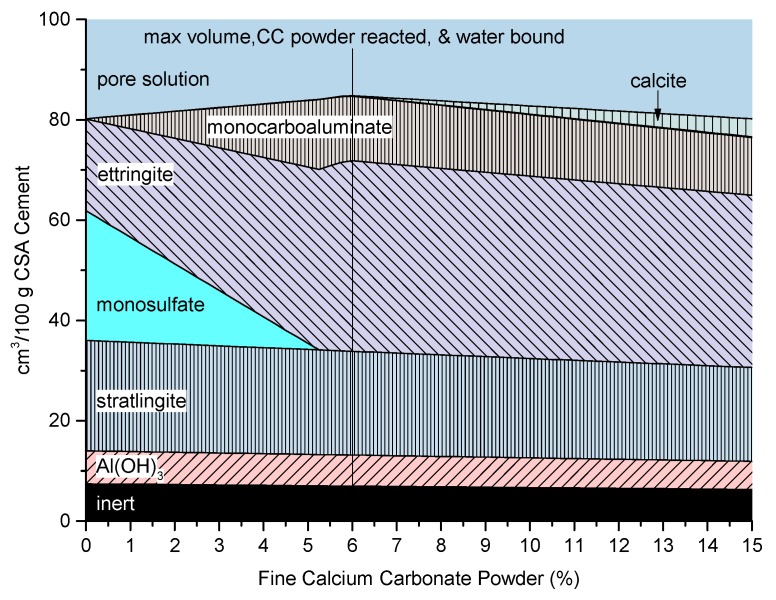
Results of modeling cement hydration with different CC contents under conditions of full hydration.

The thermodynamic modeling results of the CSAB cement with 10 wt % gypsum combined with varying amounts of fine CC powder are presented in Figure 8. As calcium carbonate is added to the system, monosulfate declines while ettringite and monocarboaluminate are formed. The monosulfate is depleted at 5.25 wt % CC powder; however, the solid volume, amount of bound water, and CC powder reacted peak at 6 wt % fine CC Powder. Between 5.25 and 6 wt % CC powder, the monocarboaluminate declines slightly while additional SO_4_–CO_3_^2−^-solid solution ettringite is formed. A similar brief decline in monocarboaluminate upon the depletion of monosulfate with additional ettringite formation can be seen in the thermodynamic modeling of a different CSA cement with calcium carbonate additions done by Martin et al. [13]. Because ettringite binds more water than monocarboaluminate, this results in a slight increase in the solid volume. Beyond 6 wt % CC powder, the calcium carbonate only acts as a filler in the CSAB cement with 10 wt % gypsum; however, by lowering the amount of added sulfate in the system, more carbonate can react [7,13,53]. The Mg from the dolomite impurity in the CC powder results in a trace of OH-hydrotalcite formation (0.14 cm^3^ at 15 wt % CC_F).

## 4. Conclusions

This study explored the hydration characteristics and material properties of CSAB cements blended with CC powder of varying fineness, and also investigated the effect of changing the gypsum content for a given CC powder content. The key findings from the conducted experiments are summarized as the following:

1. The presence of calcium carbonate powder accelerated the hydration of calcium sulfoaluminate-belite cement regardless of its fineness. The degree of acceleration became larger with increasing fineness of the calcium carbonate powder due to increased surface area of finer CC powder providing more nucleation sites for hydration product to form. On the other hand, increased gypsum content delayed the hydration at early ages.
2. The use of calcium carbonate powder having a similar fineness as the clinker decreased the total heat of hydration released due to the reduction in anhydrous clinker phases (dilution effect). However, finer calcium carbonate powder compensated for the dilution effect by accelerating the hydration through increased nucleation sites, as evidenced by the pastes’ increased total heat of hydration and acceleration of the second peak in heat evolution.
3. The calcium carbonate powders took part in the hydration of calcium sulfoaluminate-belite cement regardless of their fineness and gypsum content, forming hemicarboaluminate and monocarboaluminate, which could potentially decrease the paste’s porosity and increase its strength with additional ettringite formation.
4. Nevertheless, the strength improvement was only observed in the cases where finer calcium carbonates than the clinker were used. This implies that the positive influence of forming hemicarboaluminate and/or monocarboaluminate with additional ettringite might be diluted by the reduction in clinker components. The use of finer calcium carbonate provides more surface area for nucleation at early ages and faster dissolution due to more surface area at later ages, helping it to overcome the negative effect of diluting the clinker phases.
5. Increased gypsum content contributed to strength improvement while total early-age heat of hydration decreased due to the dilution of the clinker phases.
6. The compressive strength of the pastes showed a clear inverse relationship with the total pore volumes, indicating that total pore volume was a key factor for determining the strength of calcium sulfoaluminate-belite cements.
7. Thermodynamic modeling of calcium sulfoaluminate-belite cement hydration depending on varying amounts of calcium carbonate powder presented that the total solid volume of the cement increased with increasing calcium carbonate powder up to 6 wt %, implying that calcium carbonate amounts used in this study were possibly helpful to improve the strength and reduce the pore volume of the cements. Nevertheless, as confirmed by conducted experiments, only finer powders contributed to improving the compressive strength and reducing the pore volume.

## Figures and Tables

**Table 1:** Oxide chemical compositions of materials utilized; N.D. implies ‘not detected’.

Formula	Content (wt %)			
	CSAB	CC_F	CC_M	CC_C
CaO	44.6	57.6	58.7	55.8
Al_2_O_3_	32.8	N.D.	N.D.	0.4
SiO_2_	8.7	N.D.	0.4	0.3
SO_3_	7.7	N.D.	N.D.	N.D.
Fe_2_O_3_	2.1	0.1	0.1	0.1
MgO	2.0	1.8	2.6	2.6
TiO_2_	1.4	N.D.	N.D.	N.D.
K_2_O	0.4	N.D.	N.D.	N.D.
ZrO_2_	0.1	N.D.	N.D.	N.D.
LOI	0.3	40.3	37.9	40.8

**Table 2:** Quantitative XRD results of CSAB clinker and all CC powders; N.D. implies ‘not detected’.

Phases	Mineral Name	Rietveld Quantification (wt %)			
		CSAB	CC_F	CC_M	CC_C
C_4_A_3_$\bar{S}$	Ye’elimite	68.2	N.D.	N.D.	N.D.
β-C_2_S	Larnite	19.6	N.D.	N.D.	N.D.
C_2_AS	Gehlenite	3.0	N.D.	N.D.	N.D.
CT	Pervoskite	4.0	N.D.	N.D.	N.D.
M	Periclase	1.3	N.D.	N.D.	N.D.
C_4_AF	Brownmillerite	3.8	N.D.	N.D.	N.D.
C$\bar{C}$	Calcite	N.D.	96.9	95.2	88.1
CM$\bar{C}$_2_	Dolomite	N.D.	3.1	4.8	11.9
SUM		99.9	100	100	100

**Table 3:** Detailed mixture proportions in weight ratios.

Label	CSAB	Gypsum	Calcium Carbonate			Water	Gypsum/Ye’elimite Molar Ratio (*m*)
			CC_F	CC_M	CC_C		
CSAB_g0.1_0	89.5	10.5	0.0	0.0	0.0	50.0	0.65
CSAB_g0.1_F0.05	85.0	10.0	5.0	0.0	0.0	50.0	0.65
CSAB_g0.1_M0.05	85.0	10.0	0.0	5.0	0.0	50.0	0.65
CSAB_g0.1_C0.05	85.0	10.0	0.0	0.0	5.0	50.0	0.65
CSAB_g0.15_M0.05	80.0	15.0	0.0	5.0	0.0	50.0	1.04
CSAB_g0.2_M0.05	75.0	20.0	0.0	5.0	0.0	50.0	1.47
